# Current Opinions on Chemoresistance: An Overview

**DOI:** 10.6026/97320630014080

**Published:** 2018-02-28

**Authors:** Saba Hasan, Ruba Taha, Halima El Omri

**Affiliations:** 1 Medical Oncology Department, National Center for Cancer Care and Research, Hamad Medical Corporation, Doha, Qatar.

**Keywords:** Chemoresistance, CSCs, drug efflux, biomarkers, tumor cells

## Abstract

Sub population of cancer cells, referred to as Cancer stem cells (CSCs) or tumor initiating cells, have enhanced metastatic potential that drives tumor progression. CSCs have been found to hold intrinsic resistance to present chemotherapeutic strategies. This resistance is attributed to DNA reparability, slower cell cycle and high levels of detoxifying enzymes. Hence, CSCs pose an obstacle against chemotherapy. The increasing prevalence of drug resistant cancers necessitates further research and treatment development. The current review presents the essential mechanisms that impart chemoresistance in CSCs as well as the epigenetic modifications that can induce drug resistance and considers how such epigenetic factors may contribute to the development of cancer progenitor cells, which are not killed by conventional cancer therapies.

## Background

Cancers develop in complex tissue environments, which they depend upon for sustained growth, invasion and metastasis. Interactions between tumor cells and the associated stroma represent a powerful relationship that influences disease initiation, progression and patient prognosis. Whereas cancer had previously been viewed as a heterogeneous disease involving aberrant mutations in tumor cells, it is now evident that tumors are also diverse by nature of their micro environmental composition, and stromal cell proportions or activation states [1]. Tumor cells gradually leave the primary tumor and enter the circulation. Once there, they are called circulating tumor cells (CTCs). Circulating tumor cells must overcome a number of physiological hurdles to disseminate. To enter the circulation, it is essential that the tumor cells must invade from the epithelium or tumor of origin, navigate through their local microenvironment, and traverse the endothelium (intravasation). Once in circulation, CTCs are bound to tolerate and survive immunological pressures, exit from circulation (extravasation), and successfully incorporate within their new tissue. Circulating tumor cells with mesenchymal features predict poor outcome in a number of cancers, indicating that this phenotypic shift provides an advantage in circulation and/or distant sites [2].

Spread of cancer depends on the detachment of aggressive malignant cells from the primary tumor into the bloodstream as a principal source of the further metastasis [3]. It is known that Circulating Tumor Cells (CTCs) acquire the ability to evade the host immune system and to reach a distant organ, usually the liver in CRC, where they efficiently establish a secondary tumor growth site [4, 5]. Circulating tumor cells (CTCs) are tumor cells shed from primary and metastatic sites that circulate in the peripheral blood and can be detected by many advanced methods. The cells are present in patients with distant metastases, and with early, localized tumors. The development of personalized treatment for cancer patients depends on the specification of the molecular character of their disease. Therefore, it is essential to monitor the mechanism of resistance in tumor growth [6].

Metastasis is a biologically complex process consisting of numerous speculative events that may differ across various cancer types. CTCs bear a tremendous potential to improve our understanding of steps involved in the metastatic cascade, starting from intravasation of tumor cells into the circulation until the formation of clinically detectable metastasis [7]. Circulating tumor cells (CTCs) in the blood and disseminated tumor cells (DTCs) that have already reached a secondary organ, but have not yet grown to become clinical overt metastasis, are frequently detected in patients, thus linking to poor prognosis [8]. It is evident that tumor is heterogeneous in nature and that certain cells have increased tumor-initiating abilities. These tumor-initiating cells are also referred to as cancer stem cells (CSCs) and are hypothesized to self-renew (maintaining a population of CSCs) and to differentiate into less tumorigenic Non-CSCs [9]. Overall, there are 2 putative mechanisms by which chemoresistance may arise in cancer Chemoresistance in cancer is caused by (1) Therapy-induced molecular alterations and (2) The presence of cellular heterogeneity within the tumor bulk

### Drug Resistance in Cancer Stem Cells

**Figure 1:**
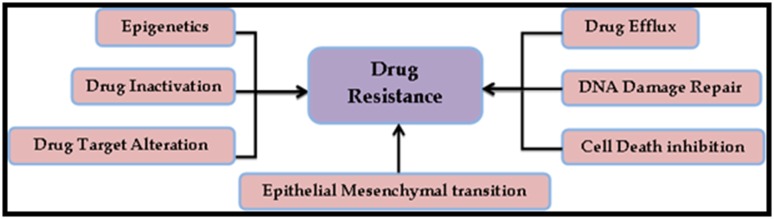
Mechanisms that promote direct or indirect drug resistance in human cancer cells. These mechanisms can act independently or in combination and through various signal transduction pathways [10].

The success of most chemotherapeutics relies on the drug's ability to decrease tumor size or induce short-term remission. This measure of success is intuitive and many drugs evaluated by these criteria are used in effective chemotherapeutic regimens. Still, it is evident that in few cases, eliminating the bulk of cancer cells may effectively select for resistant cells. Cancer cells may acquire resistance to chemotherapy, or may have a high basal level of resistance through a variety of mechanisms (Figure 1). Cancer cells often have defective DNA repair pathways, and due to rapid proliferation, these cells are often in S-phase, which is a vulnerable phase for DNA damage. When the DNA repair cascades are unable to fix the damage, cell-cycle checkpoint components are activated which can recruit additional DNA repair components or activate apoptosis [9].

Cancers possess the ability to develop resistance to traditional therapies, and increasing prevalence of these drug resistant cancers necessitates advanced research and development of active treatment strategies. Drug resistance develops as a result of tolerance to pharmaceutical treatments. This concept was firstly discovered in antibiotic resistant bacteria. Since then, similar mechanisms have been found to occur in many diseases, including cancer. Some methods of drug resistance are disease specific, while others, such as drug efflux, which is observed in microbes and human drug-resistant cancers, are evolutionarily conserved. Although many types of cancers are initially susceptible to chemotherapy, but, over the time they can develop resistance through various mechanisms, such as DNA mutations and changes in metabolism that promote drug inhibition and degradation.

### Drug Inactivation

Drug activation in vivo involves complex mechanisms where different proteins interact with specific substances. These interactions lead to modification, partial degradation, or complexing the drug with other molecules or proteins, ultimately leading to its activation. Many anticancer drugs must undergo metabolic activation in order to acquire clinical efficacy. However, cancer cells may develop resistance to such treatments through decreased drug activation. One example of this is observed in the treatment of acute myelogenous leukemia with cytarabine (AraC), a nucleoside drug that is activated after multiple phosphorylation events that convert it to AraCtriphosphate [11, 12]. Another important example of drug activation and inactivation is observed in the GST superfamily, which is a group of detoxifying enzymes that function to protect cellular macromolecules from electrophilic compounds. GSTs assist in the development of drug resistance through direct detoxification and inhibition of the mitogen-activated protein kinase (MAPK) pathway [13].

### Alteration of Drug Targets

Efficacy of any drug is influenced by its molecular target and alterations of this target by mutations or modifications of expression levels. Target alterations in cancers can ultimately lead to drug resistance. For example, topoisomerase II, an enzyme that prevents DNA from becoming super coiled is an essential target for certain anticancer drugs. Aditionally, drug resistance is also achieved by alteration in the signal transduction pathway that mediates drug activation. For example, the treatment of HER 2-positive breast cancer tumors with trastuzumab (Herceptin), a humanized monocolonal antibody, has had high levels of efficacy in combination with chemotherapy. However, many patients who initially respond to trastuzumab develop resistance and relapse, despite continued treatment. Trastuzumab also has limited efficacy as a single agent, and some patients do not respond to treatment at all, despite being HER2-positive. The mechanism of resistance is thought to be associated with cell cycle inhibition, co-expression of growth factor receptors, activation of PI3K/Akt pathway, and loss of phosphatase and tensin homolog (PTEN) function [14, 15].

### Drug Efflux

It is one of the most extensively studied mechanisms of cancer drug resistance and specifically involves reduction of drug accumulation by enhancing efflux. ABC transporters are transmembrane proteins present in human cells as well as all extant phyla. They function to transport a variety of substances across cellular membranes. Members of the ATP-binding cassette (ABC) transporter family proteins are essential regulators in plasma membrane of healthy cells and enable this efflux. Though a transporter's structure varies from protein to protein (e.g., there are 49 known members of the ABC family in humans), they are all classified by the presence of two distinct domains-a highly conserved nucleotide binding domain and a more variable transmembrane domain [16]. Binding of a substrate to trans membrane domain leads to ATP hydrolysis at the nucleotide binding site, which drives a conformational change that pushes the substrate out of the cell. This efflux plays an important role in preventing over accumulation of toxins within the cell [17]. ABC transporters are highly expressed in the epithelium of the liver and intestine, where the proteins protect the body by pumping drugs and other harmful molecules into the bile duct and intestinal lumen. They also play a major role in maintaining the blood-brain barrier [18, 19].

### DNA Damage Repair

The repair of damaged DNA has an essential role in anticancer drug resistance. In response to chemotherapeutic drugs that cause direct or indirect damage to DNA, it is seen that DNA damage response (DDR) mechanisms can reverse the drug induced damage. For example, platinum-containing chemotherapy drugs such as Cisplatin cause harmful DNA crosslinks, leading to apoptosis. However, resistance to platinum based drugs often arises due to nucleotide excision repair and homologous recombination, the primary DNA repair mechanisms involved in reversing platinum damage [20, 21, 22]. Thus, the efficacy of DNA-damaging cytotoxic drugs relies on the failure of the cancer cell's DDR mechanisms. Repair pathway inhibition in conjunction with DNA damaging chemotherapy may sensitize cancer cells and therefore enhance efficacy of the therapy.

### Cell Death Inhibition

Cell death by apoptosis and autophagy are two important regulatory events. Though they are antagonistic to each other, yet, they contribute to cell death. Apoptosis has two established pathways: an intrinsic pathway mediated by the mitochondria that involve B-cell lymphoma 2 (BCL-2) family proteins, caspase- 9 and Akt, and an extrinsic pathway that involves death receptors on the cell surface. The intrinsic and extrinsic pathways merge through the activation of down-stream caspase-3, which ultimately causes apoptosis. However, there is also additional cross-linking between the pathways. Recombinant forms of tumor necrosis factor related apoptosis-inducing ligand (TRAIL) and agonistic antibodies to these receptors could induce apoptosis through the activation of caspase-8. Many cancer drugs also induce apoptosis via the activation of c-Jun N-terminal kinases (JNK), which is downstream of the MAPK pathway. These results suggest that cancer cells, including those, which are drug resistant, can be effectively treated by using one drug that makes the cells susceptible to death through the altered expression or regulation of cell death pathway members in combination with another cytotoxic drug that kills the cells in their vulnerable states.

### Epithelial-Mesenchymal Transition and Metastasis

The role of EMT in cancer drug resistance is an emerging area of research. The epithelial to mesenchymal transition (EMT) represents the mechanism by which solid tumors become metastatic. Metastasis is a complex phenomenon that includes changes in a cancer cell and the stromal cells that make up its environment. It includes angiogenesis, i.e. the formation of new blood vessels around metastatic tumors. During EMT, tumor cells reduce the expression of cell adhesion receptors, which help in cell-cell attachment, including integrins and cadherins, and increase the expression of cell adhesion receptors that induce cell motility. Cell motility also relies on cytokines and chemokines, which may be released either by tumor cells or by cells in their microenvironment. Additionally, higher expression of metallo proteases on the surface of tumors eases the outward movement of cells, promoting metastasis [23, 24].

Drug resistance in cancer cells may also develop during the signaling processes of differentiation, which are essential for EMT. For example, the increased expression of integrin avβ1 in colon cancer positively regulates transforming growth factor β (TGFβ) expression, required for EMT, which further serves as a survival signal for cancer cells against drugs [25]. Integrin avβ1 interacts with stromal cell adhesion molecules to convey such signals. Similarly, β3 integrin and src regulate TGFβ mediated EMT in mammary cancer. Ligation of integrin β1 provides proliferative and survival signal-mediated FAK kinase in lung cancers.

### Drug biomarker development in oncology

The acquisition of tumor resistance to chemotherapies, observed in virtually all cases, significantly limits their utility, and remains a substantial challenge to the clinical management of advanced cancers. Multidrug resistance may be intrinsic or acquired during treatment, arising from genetic mutations, tumor microenvironment pH changes, activation of survival signaling pathways, increased drug efflux through the ABC transporter proteins, or the selection and emergence of an inherently resistant subpopulation of tumor cells [26, 27, 28, 29, 30].

**Figure 2:**
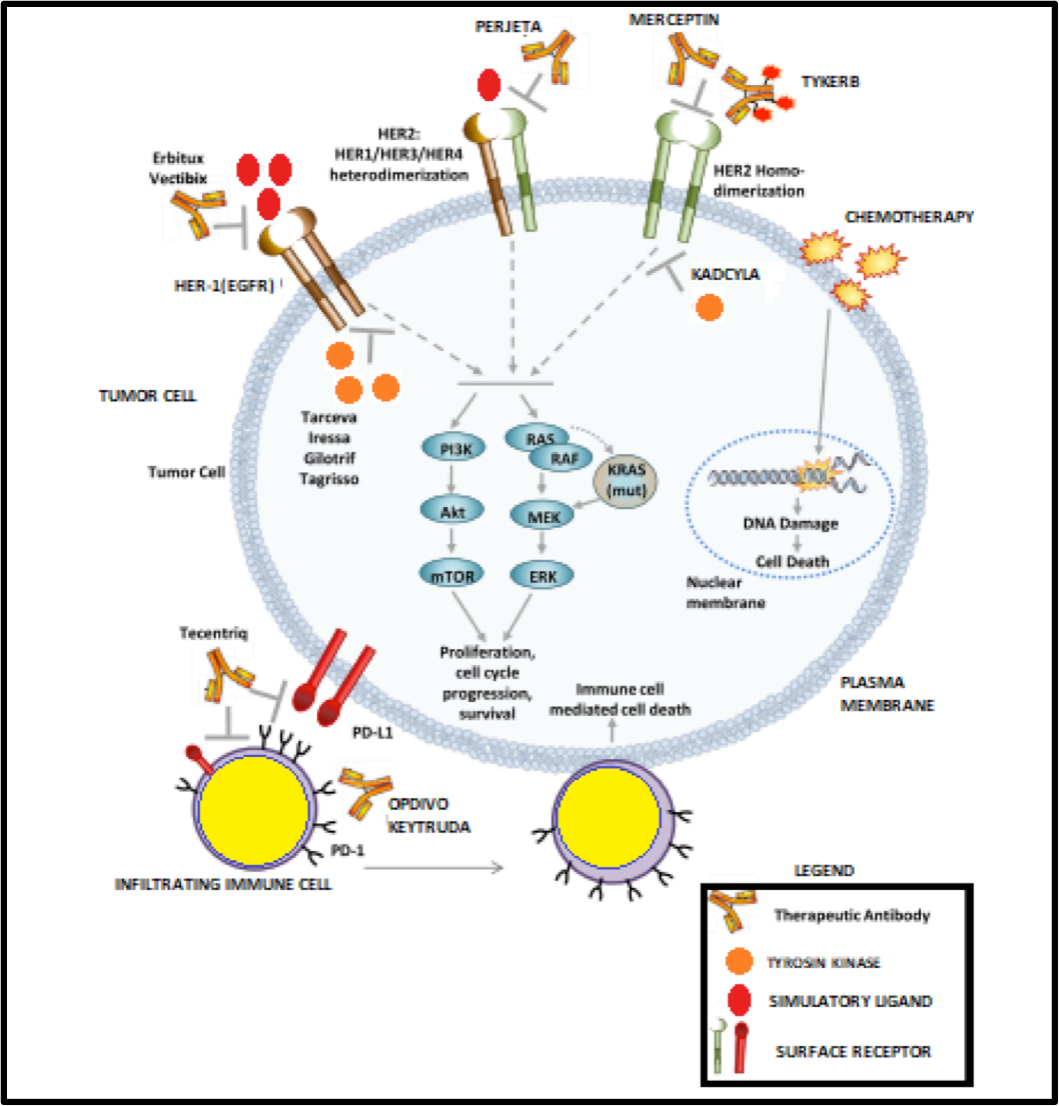
Systematic view of therapeutics targeting HER2, EGFR or PD-1/PD-L1

Improvement of cancer treatment outcomes is possible through the development of molecularly targeted therapeutics that block or stimulate specific-signaling pathways of tumor cells. Over the past two decades, the US Food and Drug Administration (FDA) have approved more than 80 molecularly targeted oncology drugs for treating various human malignancies. These targeted therapies include small molecules and monoclonal antibodies aimed to block specific pathways that lead to carcinogenesis and tumor growth. They have multiple modes of action: inducing programmed cell death (apoptosis) of cancer cells, blocking specific enzymes and growth factor receptors involved in cancer cell proliferation, or modifying the function of proteins that regulate gene expression and other cellular functions. Signaling components of human epidermal growth factor receptor 2 (HER2), epithelial growth factor receptor (EGFR), and programmed death receptor-1 (PD-1) are among these therapeutic targets that have led to successful development of molecular marker-driven cancer therapy (Figure 2). The targeted therapies appear considerably promising for improved patient outcomes by selective action on specific oncogenic proteins.

Research aimed at characterizing the molecular signatures along the cancer progression continuum could inform the codevelopment of targeted therapy and predictive biomarker in a number of ways (1) enhancement of predictions about therapeutic efficacy or drug resistance, (2) revelation of new potential mechanisms of drug resistance, and (3) acceleration for developing of next-generation targeted therapeutics that bypass the potential resistance mechanisms.

### Case Report

A rare case report of chemoresistant Gestational Trophoblastic Neoplasia (GTN) confirmed to be Placental Site Trophoblastic Tumour (PSTT) is explained here in order to understand the phenomena of chemoresistance [31]. In June 2011, a 24-year-old woman, in her 4th month of her gestation, history of passage of grape like mass and also had history of vaginal spotting in her first trimester. After Ultrasonography (USG), she showed bulky uterus with molar pregnancy. She underwent ultrasound guided suction evacuation. Histopathology examination of specimen reported as molar pregnancy.

On 24th Feb. 2012, i.e. eight months following evacuation, the patient presented with history of vaginal bleeding for 13 days following regular cycles after evacuation and her last menstrual period was Feb 11th 2012, there was no evidence of pregnancy. Per speculum and bimanual examinations revealed congested cervix with mucoidal discharge, uterus soft in consistency, right fornix tenderness present. The striking rise of serum β hCG level to 11,203 mIU/ml was noted. USG scan showed a well-defined hyper echoic lesion measuring 2.5*2.2*2 cm with few areas of heteroechogenecity in the center. Patient was started on first line of chemotherapy with injection methotrexate 50 mg intramuscular weekly for total five cycles with folinic acid rescue at 21 days interval from March 2012 to May 2012. The β hCG levels were variable as In June 2012, the patient was then started on second line chemotherapy with course of injection Dactinomycin, 12mcg/kg for five days. But even after six weeks course of chemotherapy, the hCG level was still high, so then patient was again put on with two cycles of chemotherapy with injection cyclophosphamide 600mg/m2 intravenously in saline over 30 minutes and injection Vincristine 1mg/m2 intravenously bolus over one minute given 21days interval. So, during the whole course of chemotherapy, there was a variable rise and fall of β hCG level. Ultimately, the patient was advised total abdominal hysterectomy on September 2012. The HPE report was suggestive of PSTT. This explains the low chemosensitivity behaviour of the tumour. The report is presented here because of the challenges faced during the course of the treatment process and its rare occurrence following molar pregnancy [31].

Another rare case of a chemoresistant invasive mole of the uterus, which developed following the evacuation of a molar pregnancy, has been reported [32]. 28 years old Female, gravida three para one living one abortion one with previous ceasearian section had chief complaints of two months of amenorrhea with bleeding per vaginum since one day and with ultrasonography report suggestive of vesicular mole. Gestational trophoblastic neoplasia, 60% was secondary to hydatidiform mole, 30% to abortion, and 10% secondary to full term pregnancy or ectopic pregnancy. Chemotherapy of patient on single agent was started. After one cycle serum beta hcg was repeated and was 225000 iu /ml. Patient was started on EMACO REGIMEN. Three cycle of EMACO regimen were undertaken. There was no significant decrease in size of lesion and serum b hcg level even after three cycles of EMACO regimen. Hence tough decision hysterectomy was done in view of chemoresistant invasive persistent trophoblastic disease. The case report emphasizes that persistent trophoblastic disease needs to be defined precisely and early diagnosis and treatment. Chemoresistant invasive mole surgical intervention at proper time in management of persistent trophoblastic disease is the key to 100% survival in gestational trophoblastic neoplasia. The following Table 1 given below indicates how cellular metabolism can be targeted to improve cancer therapeutics.

## Conclusion

Cancer drug resistance is a complex phenomenon influenced by drug inactivation, drug target alteration, drug efflux, DNA damage repair, cell death inhibition, EMT, inherent cell heterogeneity, epigenetic effects, or any combination of these mechanisms. The current paradigm states that combination therapy should be the best treatment option because it should prevent the development of drug resistance and be more effective than any one drug on its own [40, 41, 42, 43, 44]. Therefore, such treatment regimens should be considered and developed to counteract the increasing prevalence of drug resistance in cancers. Cancer progenitor cells are often drug resistant as well. These progenitor cells can persist in patients seemingly in remission, and they are able to remain stationary or migrate to other sites during metastasis. Thus, cancer progenitor cells can cause cancer relapse at the original tumor site or in distant organs. The next step in anticancer therapy development should target the elimination of such cancer progenitor cells. The existence of a small population of drug resistant cancer cells poses another complexity that is difficult to address [45]. These drug resistant cancer cells also contribute to cancer relapse after apparent remission. Insights on the mechanisms of resistance will assist in the design of more effective strategies to overcome it in cancer cells and tumors. Therefore, it is important to build on existing knowledge related to tumor heterogeneity and potential mechanisms of targeted therapy evasion. Conceivably, tumour heterogeneity may impede the identification of predictive biomarkers, and the quest for personalised, or even curative treatment, and is an area of cancer research worthy of collaborative effort.

## Figures and Tables

**Table 1:** Targeting cellular metabolism improves cancer therapeutics

Targeted metabolism	Targeted metabolic enzymes	Metabolic inhibitors	Cancer therapeutics	Cancer types (in vitro / in vivo)	Reference
Glycolysis	GLUT1	Phloretin	Daunorubicin	Colon cancer (in vitro), Leukemia (in vitro)	[33]
	GLUT4	Ritonavir	Doxorubicin	Multiple myeloma (in vitro)	[34]
	HK	3-BrPA	Prednisolone	Leukemia (in vitro)	[35]
Citric acid cycle	PDK3	siRNA	Patlitaxel	Cervical Cancer	[36]
	PDK	DCA	Omeprazole	Fibrosarcoma (in vitro and in vivo)	[37]
Fatty acid synthesis	FASN	Cerulenin	Docetaxel	Breast cancer (in vitro)	[38]
		Orlistat	Adriamycin	Breast cancer (in vitro)	[39]
